# FNDC4 Inhibits RANKL-Induced Osteoclast Formation by Suppressing NF-*κ*B Activation and CXCL10 Expression

**DOI:** 10.1155/2018/3936257

**Published:** 2018-05-30

**Authors:** Zheng-tao Lv, Shuang Liang, Kun Chen, Jia-ming Zhang, Peng Cheng, Jia-chao Guo, Qing Yang, Chen-he Zhou, Hui Liao, An-min Chen

**Affiliations:** ^1^Department of Orthopedics, Tongji Hospital, Tongji Medical College, Huazhong University of Science and Technology, Wuhan 430030, China; ^2^Department of Orthopedic Surgery, Second Affiliated Hospital, School of Medicine, Zhejiang University, Hangzhou, China

## Abstract

FNDC4 acts as an anti-inflammatory factor on macrophages and improves mouse model of induced colitis. Considering osteoclast formation is characterized by the activation of inflammation-related pathways, we thus speculated that FNDC4 may play a pivotal role in this process. RT-qPCR analysis was performed to confirm the expression of osteoclast formation related genes in primary murine bone marrow macrophages (BMMs). RANKL-treated BMMs were cultured with FNDC4 to evaluate the effect of FNDC4 on osteoclast differentiation. TRAP staining and bone resorption pits assay were used to assess osteoclast formation and bone resorption, respectively. Luciferase assay and western blotting analysis were conducted to determine whether FNDC4 inhibited osteoclast formation via NF-*κ*B signaling in RAW 264.7 cells. Furthermore, to identify gene signatures in FNDC4-treated BMMs and to use these to elucidate the underlying molecular mechanisms during osteoclast formation, we adopted a bioinformatics approach by downloading the GSE76172 gene expression profiling dataset from the Gene Expression Omnibus (GEO) database. FNDC4 inhibited RANKL-induced osteoclastogenesis and mature osteoclast resorptive function in a dose-dependent manner. Results of NF-*κ*B luciferase assay suggested that FNDC4 could significantly suppress the RANKL-induced NF-*κ*B transcriptional activity. Based on the protein-protein interaction network, CXCL10 was identified as the differentially expressed gene with the highest connectivity degree (degree = 23); it was drastically downregulated in the presence of FNDC4, but supplementation of CXCL10 (10 ng/mL) partially ameliorated the FNDC4-induced inhibition of osteoclast formation. Taken together, we speculated that FNDC4 could suppress osteoclast formation via NF-*κ*B pathway and downregulation of CXCL10.

## 1. Introduction

Bone is a dynamic organ that undergoes continuous remodeling throughout life; its homeostasis is regulated by osteoblast-mediated bone formation and osteoclast-mediated bone resorption [1]. The balance between osteoclasts and osteoblasts is critical to the strength and integrity of bone. Enhanced osteoclast activation and the associated excessive bone resorption lead to several osteolytic bone diseases, such as osteoporosis, rheumatoid arthritis, periodontitis, and Paget's disease of bone [2, 3].

Osteoclasts are multinucleated giant cells originating from the monocyte/macrophage lineage of hematopoietic stem cells. Osteoclasts are formed through cell fusion, and the process is mainly regulated by two essential cytokines, macrophage colony stimulating factor (M-CSF) and receptor activator of nuclear factor-*κ*B ligand (RANKL) [4, 5]. M-CSF is responsible for maintaining the proliferation and survival of osteoclast progenitor cells, whereas RANKL initiates osteoclast precursors to differentiate into mature osteoclasts by activating its receptor RANK. Upon the binding of RANKL with RANK, tumor necrosis factor (TNF) receptor-associated factor 6 (TRAF6) is recruited, which in turn triggers the activation of a cascade of intracellular pathways including nuclear factor-*κ*B (NF-*κ*B), mitogen-activated protein kinases (MAPK), nuclear factor of activated T cells cytoplasmic 1 (NFATc1), and activator protein 1 (AP-1) [6–8]. Among these signaling molecules, NFATc1 is a core RANKL-mediated transcription factor; it regulates osteoclast differentiation by inducing the expression of diverse osteoclastogenesis-related genes such as Cathepsin K (CtsK) and tartrate-resistant acid phosphatase (TRAP) [8, 9].

FNDC4 is a secreted factor sharing high homology with the exercise-induced myokine irisin [10, 11]. Irisin is secreted as a cleavage molecule from fibronectin type III domain containing 5 (FNDC5) expressed on plasma membrane of myocytes and, to some extent, adipocytes [12]. It has been reported that irisin could directly target osteoblast and promote osteoblast proliferation and differentiation via p38/MAPK signaling* in vitro* [13]. Furthermore, irisin, released upon physical activity, displays anabolic actions on skeleton by increasing cortical bone mass and bending strength* in vivo* [14]. However, no published studies have addressed the anabolic effect of FNDC4 on skeletal system. Bosma and coworkers [10] first characterized the immunomodulating properties of FNDC4. In the mice model of induced colitis, FNDC4 treatment could markedly reduce inflammation and ameliorate disease severity, suggesting the potential therapeutic effect. Moreover, FNDC4 prominently binds to macrophages and leads to a state of dampened macrophage activity, supported by downregulation of inflammatory gene expression (such as CXCL9, CXCL10, Tgfb1, and Csf1). Bosma et al. [10] attributed the therapeutic effect of FNDC4 in colitis to the inhibitory effect on macrophages. As osteoclast differentiation is well characterized by the activation of inflammation-related pathways, we speculated that FNDC4 might play a certain role in this process.

Therefore, we performed this* in vitro* study with the aim of investigating the potential role of FNDC4 in the inhibition of osteoclast formation and bone resorbing function and attempting to elucidate the underlying molecular mechanism using a bioinformatics approach.

## 2. Methods and Materials

### 2.1. Reagents and Antibodies

Recombinant soluble murine M-CSF (315-02), RANKL (315-11), and CXCL10 (250-16) were purchased from PeproTech (Rocky Hill, NJ, USA). Primary antibodies against mouse P65 (8242), p-P65 Ser536 (3033), I*κ*B-*α* (4814), and p-I*κ*B-*α* Ser32 (2859) used in western blots were obtained from Cell Signaling Technology (Beverly, MA, USA). Rabbit polyclonal antibody against CXCR3 (ab181013) was purchased from Abcam (Cambridge, MA, USA). Mouse antibody against *β*-actin (BM5180) was from Boster (Wuhan, China).

### 2.2. Cell Culture

The primary BMMs were collected from tibias and femurs of 6-week-old male C57BL/6 mice by flushing the bone marrow with culture medium. The isolated nonadherent purified BMMs were cultured in *α*-MEM supplemented with 10% fetal bovine serum (FBS), 100 IU/mL penicillin, 100 IU/mL streptomycin, and 30 ng/mL of M-CSF. RAW 264.7 cells, a mouse leukemic monocyte/macrophage cell line, were purchased from the American Type Culture Collection (ATCC) and were maintained in *α*-MEM supplemented with 10% FBS, 100 IU/mL penicillin, and 100 IU/mL streptomycin. All cell cultures were maintained at 37°C in a humidified 5% CO_2_ atmosphere.

### 2.3. Cytotoxicity Assay

To determine the effect of FNDC4 on the cell viability, cytotoxicity assay was conducted using the CCK-8 according to the manufacturer's instruction. Primary BMMs were seeded in 96-well plate at a density of 3000 cells per well and incubated with M-CSF (30 ng/mL). After 24 hours' culture, BMMs were treated with varying concentrations of FNDC4 in the presence of M-CSF. Cell viability and proliferation were measured on day 1, day 3, and day 5 by adding the CCK-8 reagent to the medium and incubating at 37°C for an hour. Optical density was measured at 450 nm with an Epoch microplate reader (Bio-Tek, Winooski, Vermont, USA).

### 2.4. In Vitro Osteoclast Differentiation Assay

To determine the effect of FNDC4 on osteoclast formation, BMMs were seeded in 96-well plates (10,000 cells per well) and cultured with M-CSF (30 ng/mL) and RANKL (100 ng/mL) in the presence of different concentrations of FNDC4. The culture medium was replaced every day. After 5 days' induction, cells were fixed using 4% paraformaldehyde PBS for 10 minutes at room temperature and then stained for TRAP according to the manufacturer's protocol. TRAP-positive multinucleated cells with more than three nuclei were counted as osteoclasts.

### 2.5. Resorption Pit Assay

To assess the bone resorbing function of mature osteoclasts, BMMs were seeded in a Corning Osteo Assay Surface plate (Corning Incorporated Life Science, New York, USA) with RANKL (100 ng/mL) and M-CSF (30 ng/mL), in the presence of different concentrations of FNDC4 (100 ng/mL, 500 ng/mL, and 1000 ng/mL). The medium was changed once per day. After the incubation at 37°C for 7 days, cells were washed with bleach solution. Images of pit formation were obtained and quantified.

### 2.6. RNA Isolation and Real Time Quantitative PCR Analysis

RT-qPCR was performed as previously described [15]. Total RNA was extracted from BMMs using the commercially available AZfresh reagent (Azanno Biotech, Sweden) following the manufacturer's instructions. The concentration of RNA was determined and cDNA was synthesized for subsequent qPCR using the total RNA with the ReverTra Ace qPCR RT Kit (Toyobo, Japan). cDNA was amplified using SYBR Green (Toyobo, Japan) with the specific primers. The specific mouse prime sequences used in this study were as followed: TRAP (forward: TACCTGTGTGGACATGACC; reverse: CAGATCCATAGTGAAACCGC); CtsK (forward: TGTATAACGCCACGGCAAA; reverse: GGTTCACATTATCACGGTCACA); NFATc1 (forward: CAACGCCCTGACCACCGATAG; reverse: GGGAAGTCAGAAGTGGGTGGA); CXCL10 (forward: CCAAGTGCTGCCGTCATTTTC; reverse: GGCTCGCAGGGATGATTTCAA); GAPDH (forward: CTCCCACTCTTCCACCTTCG; reverse: TTGCTGTAGCCGTATTCATT). GAPDH RNA was used as an internal control to normalize the data. RT-qPCR was performed using 2 *μ*g of corresponding cDNA in a 20 *μ*L reaction system. The cycling parameters were used as follows: 40 cycles of denaturation at 95°C for 15 s and annealing at 60°C for 60 s. Triplicated experiments for each sample were performed; quantification was conducted via the comparative cycle threshold (Ct) method.

### 2.7. Western Blot

Western blotting analysis was performed as previously described [16]. Briefly, BMMs pretreated with different concentrations of FNDC4 were rinsed with cold PBS and total cellular lysates were prepared using the ice-cold RIPA buffer (Boster, Wuhan, China) supplemented with protease and phosphatase inhibitor cocktail (Cell Signaling Technology, Beverly, MA, USA). The protein concentration was measured using the BCA Protein Assay kit (Beyotime, Shanghai, China) according to the manufacturer's instructions; then protein samples (20 *μ*g per well) were separated by 12% SDS-PAGE and transferred to PVDF membrane by electrophoresis. The transferred membranes were blocked with 5% skimmed milk for nonspecific binding at room temperature for 1 hour and then probed with primary antibodies against P65, p-P65 Ser536, I*κ*B-*α*, p-I*κ*B-*α* Ser32, and *β*-actin at 4°C overnight. After washing with TBST (50 mM Tris, pH 7.6, 150 mM NaCl, and 0.1% Tween 20) three times, membranes were incubated with the HRP-conjugated secondary antibodies. Enhanced chemiluminescence (ECL) substrate and ChemiDoc™ XRS+ System with Image Lab™ Software (BIO-RAD, Hercules, CA, USA) were used to visualize the immunoreactive bands.

### 2.8. Luciferase Reporter Gene Assay

To determine the inhibitory effect of FNDC4 on NF-*κ*B activation, RAW 264.7 cells were stably transfected with luciferase reporter constructs controlled by NF-*κ*B binding promoter elements [17]. Cells plated in a 48-well plate were pretreated with different concentrations of FNDC4 (100 ng/mL, 500 ng/mL, and 1000 ng/mL) for 1 hour, followed by stimulation with RANKL (100 ng/mL) for another 8 hours. Cells were then rinsed twice with PBS and lysed with luciferase lysis buffer. Luciferase activity of NF-*κ*B was measured using a Promega Luciferase Assay system (Promega, Madison, WI, USA) according to the manufacturer's instructions.

### 2.9. Bioinformatics

The raw gene expression data of GSE76172 were downloaded from the Gene Expression Omnibus (GEO) repository by the National Center of Biotechnology Information (https://www.ncbi.nlm.nih.gov/geo/) based upon the platform of GPL11533 Affymetrix Mouse Gene 1.1 ST Array; the dataset included three BMMs samples treated with recombinant FNDC4 and three BMMs samples treated with control protein. All the raw expression data were processed for normalization using the Affymetrix package in R and Bioconductor (http://www.bioconductor.org). DEGs in FNDC4-treated BMMs samples compared to control samples were identified using the Linear Models for Microarray Analysis (Limma) package; *p* < 0.05 and |log_2_⁡FC| > 0.5 were considered as the threshold.

Gene Ontology (GO) analysis and the Kyoto Encyclopedia of Genes and Genomes (KEGG) of FNDC4-treated BMMs samples have been reported by Bosma and coworkers [10] in their previous publication; thus we did not perform the functional enrichment analysis of DEGs. In order to detect the PPIs among DEGs, we uploaded the DEGs to the Search Tool for the Retrieval of Interacting Genes (STRING, available at https://string-db.org/) and selected the DEGs with >0.4 combined protein interaction score for PPI network construction (visualized by Cytoscape 2.8 software, available at http://www.cytoscape.org/). Based on the connectivity degree analysis, only the protein with the highest degree score was selected by our present study for further functional analyses.

### 2.10. Transfection with Small Interfering RNA (siRNA)

siRNA targeting CXCR3 (Ribbio, Guangzhou, China) were transfected into BMMs in the presence of M-CSF to knock down CXCR3 mRNA expression according to the manufacturer's instruction. siRNA transfection was carried out using Lipofectamine 2000 agent (Invitrogen, Carlsbad, CA, USA). The effect of selective silencing was confirmed by western blotting; the sense of mouse siRNA-CXCR3 was as follows: 5′-GGAUUUCAGCCUGAACUUUUU-3′. After treatment with siRNA for 6 hours, 100 ng/mL RANKL was added to the medium. After 3 days, the BMMs were transfected with siRNA again. On day 5, multinucleated osteoclasts were subject to TRAP staining.

### 2.11. Statistical Analysis

All data were analyzed using GraphPad Prism 7.00; results were presented as mean (*M*) ± standard deviation (SD) with at least three independent experiments. The two-tailed unpaired *t*-test was used to test statistical significance between different groups, with the *p* value less than 0.05 considered statistically significant.

## 3. Results

### 3.1. FNDC4 Inhibited RANKL-Induced Osteoclastogenesis in a Dose-Dependent Manner

**Figure 1:**
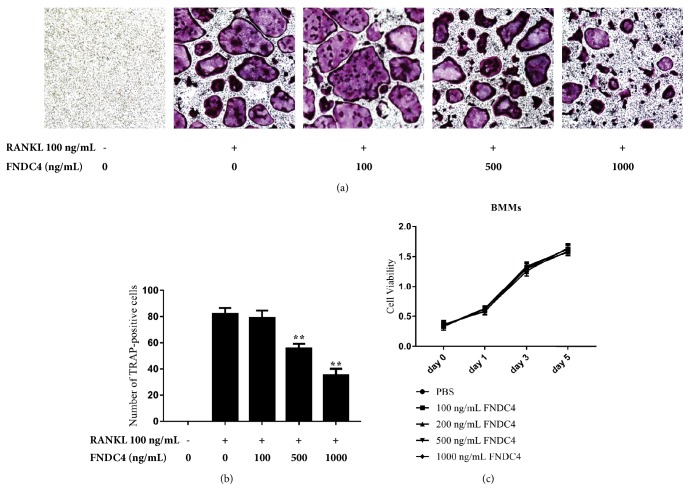
FNDC4 inhibited osteoclast formation in a dose-dependent manner. BMMs were cultured with different concentrations of FNDC4 (0, 100, 500, and 1000 ng/mL) in the presence of M-CSF (30 ng/mL) and RANKL (100 ng/mL) for 5 days. (a) Representative images of TRAP-stained cells treated with different concentrations of FNDC4 and TRAP-positive multinucleated cells with more than three nuclei were counted as osteoclasts. (b) Quantification of TRAP-positive osteoclasts showed that FNDC4 at 500 ng/mL and 1000 ng/mL could significantly reduce the number of TRAP-stained osteoclasts. (c) CCK-8 analysis was conducted to assess the effect of FNDC4 on the proliferation and viability of BMMs. All experiments were performed in triplicate and results were presented as *M* ± SD;  ^*∗*^*p* < 0.05 and ^*∗∗*^*p* < 0.01 relative to the RANKL group.

To test the effect of FNDC4 on the formation of osteoclasts, osteoclastogenesis assay was performed by stimulating BMMs with RANKL in the presence of different concentrations of FNDC4 for five days. As shown in Figures 1(a) and 1(b), in the presence of 100 ng/mL RANKL, BMMs were fully differentiated into TRAP-positive osteoclasts; the number of TRAP-positive osteoclasts was significantly reduced by the pretreatment of FNDC4 in a dose-dependent manner. The possibility that the inhibitory effect of FNDC4 on BMMs was due to cytotoxicity could be excluded because FNDC4 was found without cytotoxic effect on osteoclasts precursor cells at concentrations used in our current study (Figure 1(c)).

### 3.2. FNDC4 Attenuated Bone Resorption

**Figure 2:**
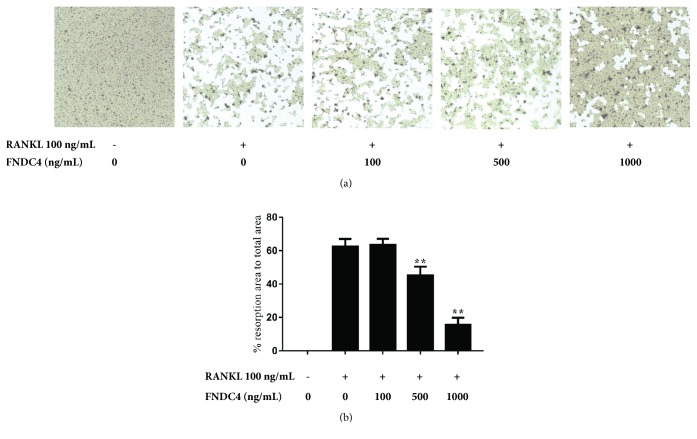
FNDC4 suppressed osteoclast function. BMMs were cultured with different concentrations of FNDC4 (0, 100, 500, and 1000 ng/mL) in the presence of M-CSF (30 ng/mL) and RANKL (100 ng/mL) for 7 days in a Corning Osteo Assay Surface plate. (a) Representative images of Osteo Assay plates after the removal of osteoclasts. The bone resorption area in the plates was measured. (b) Quantification of bone resorption area showed that FNDC4 at 500 ng/mL and 1000 ng/mL could significantly reduce the bone resorption area compared with RANKL group. All experiments were performed in triplicate and results were presented as *M* ± SD. ^*∗*^*p* < 0.05 and ^*∗∗*^*p* < 0.01 compared with the RANKL group.

To further investigate the effect of FNDC4 on osteoclast function, BMMs were seeded in a Corning Osteo Assay Surface plate with RANKL (100 ng/mL) and M-CSF (30 ng/mL), in the presence of different concentrations of FNDC4 (100 ng/mL, 200 ng/mL, 500 ng/mL, and 1000 ng/mL). Treatment of FNDC4 was shown to reduce the resorptive activity of osteoclast in a dose-dependent manner (Figure 2). At the concentration of 1000 ng/mL, FNDC4 could inhibit the bone resorbing activity of osteoclast for the most part.

### 3.3. FNDC4 Suppressed Osteoclast-Related Gene Expression

**Figure 3:**
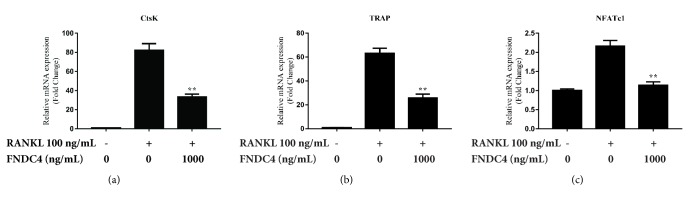
FNDC4 inhibited the expression of osteoclast-related genes (TRAP, CtsK, and NFATc1). BMMs were treated with 1000 ng/mL FNDC4 in the presence of M-CSF (30 ng/mL) and RANKL (100 ng/mL) for 2 days. (a) CtsK. (b) TRAP. (c) NFATc1. FNDC4 could significantly inhibit the RANKL-induced expression of these mRNAs. All experiments were performed in triplicate and results were presented as *M* ± SD. ^*∗*^*p* < 0.05 and ^*∗∗*^*p* < 0.01 compared with the RANKL group.

The osteoclast-related genes expressions (CtsK, NFATc1, and TRAP) that are upregulated in the response of RANKL were evaluated using RT-qPCR. As shown in Figure 3, the expression of CtsK, NFATc1, and TRAP was induced by 100 ng/mL of RANKL for 24 hours, and the addition of 1000 ng/mL FNDC4 decreased the upregulation of these RANKL-induced genes. The results of gene expression were in line with the inhibitory effect of FNDC4 on bone resorbing function as presented in Figure 2. Taken together, our data suggested that FNDC4 could inhibit bone resorption and suppress the osteoclast-specific gene expression* in vitro*.

### 3.4. FNDC4 Suppressed RANKL-Induced NF-*κ*B Activation

**Figure 4:**
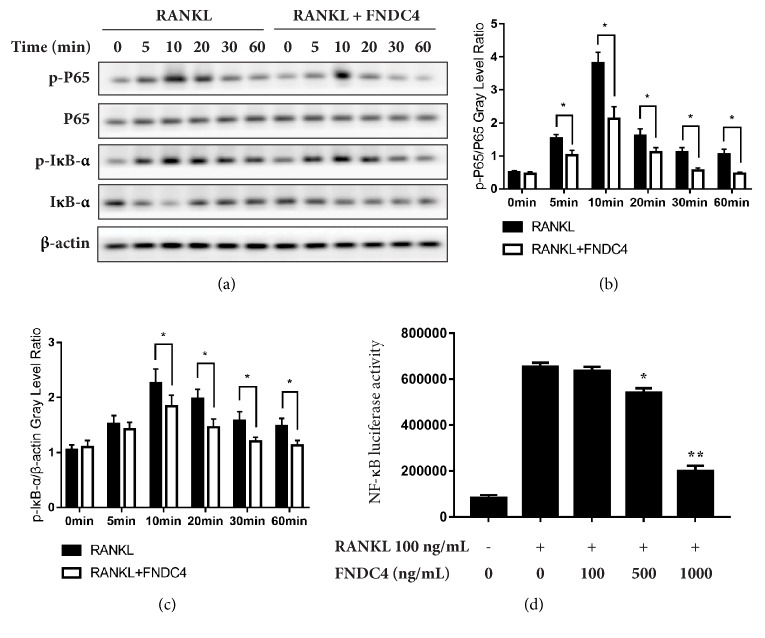
FNDC4 inhibited RANKL-induced NF-*κ*B activity. (a) BMMs were cultured with M-CSF (30 ng/mL) and RANKL (100 ng/mL) in the presence of FNDC4 (1000 ng/mL) for indicated times. The total cellular lysates were subject to western blotting analysis and immunoblotted with indicated antibodies. (b) The grey level of p-P65 was analyzed by being normalized to total P65. (c) The grey level of p-I*κ*B-*α* was analyzed by being normalized to *β*-actin. (d) RAW 264.7 cells were stably transfected with luciferase reporter constructs controlled by NF-*κ*B binding promoter elements. Cells plated in a 48-well plate were pretreated with different concentrations of FNDC4 (100 ng/mL, 500 ng/mL, and 1000 ng/mL) for 1 hour, followed by stimulation with RANKL (100 ng/mL) for another 8 hours; then the luciferase activity was measured. Data were presented as *M* ± SD. ^*∗*^*p* < 0.05 and ^*∗∗*^*p* < 0.01 compared with the RANKL group.

As NF-*κ*B pathway has been proved to play an essential role in RANKL-induced osteoclastogenesis, we thus focused on the inhibitory effect of FNDC4 on RANKL-induced NF-*κ*B transcriptional activity to gain insight into possible underlying molecular mechanisms. The luciferase assay indicated that RANKL induced a significant increase of NF-*κ*B luciferase activity compared with control; treatment of FNDC4 had an inhibitory effect on transcriptional activity of NF-*κ*B in a dose-dependent manner (Figure 4(d)).

To further confirm this finding, we performed western blotting analysis to evaluate the effect of FNDC4 on activation of NF-*κ*B and degradation of I*κ*B-*α*. In order to detect I*κ*B-*α* degradation and P65 phosphorylation, BMMs were treated with RANKL at 5 min, 10 min, 20 min, 30 min, and 60 min in the presence of absence of 1000 ng/mL FNDC4. As shown by western blotting (Figures 4(a)–4(c)), RANKL stimulation led to the phosphorylation and degradation of I*κ*B-*α* after 10 min and I*κ*B-*α* resynthesized at 30 min. However, FNDC4 treatment for 30 min followed by RANKL stimulation could suppress the degradation of I*κ*B-*α*; this finding was also supported by lower level of p-P65 at 5 min after the stimulation with both FNDC4 and RANKL than RANKL treatment alone. Collectively, FNDC4 could delay the RANKL-induced degradation of I*κ*B-*α* and attenuate the nuclear translocation of NF-*κ*B P65.

### 3.5. FNDC4 Inhibited RANKL-Induced Osteoclast Formation through Downregulation of CXCL10

**Figure 5:**
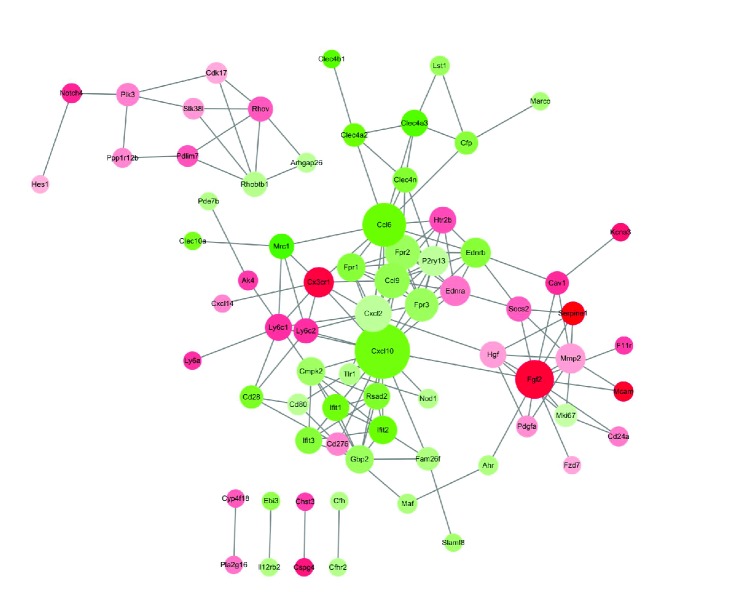
Protein-protein interaction (PPI) network of differentially expressed genes (DEGs). Red nodes and green nodes illustrate upregulated DEGs and downregulated DEGs, respectively. Color depth represents fold-change, with deeper colors indicating greater fold-change. Nodes with higher degree values are depicted with larger sizes.

To identify gene signatures in FNDC4-treated BMMs and to use these to elucidate the underlying molecular mechanisms during osteoclast formation, we adopted a bioinformatics approach by downloading the GSE76172 gene expression profiling dataset from the Gene Expression Omnibus database. GO analysis and the KEGG of FNDC4-treated BMMs samples were reported by Bosma and coworkers in their previous publication; thus we did not perform the functional enrichment analysis of DEGs. Instead, we uploaded these data to STRING and selected the DEGs with >0.4 combined protein interaction score for PPI network construction (Figure 5). According to the connectivity degree, CXCL10 was selected for further functional analysis.

**Figure 6:**
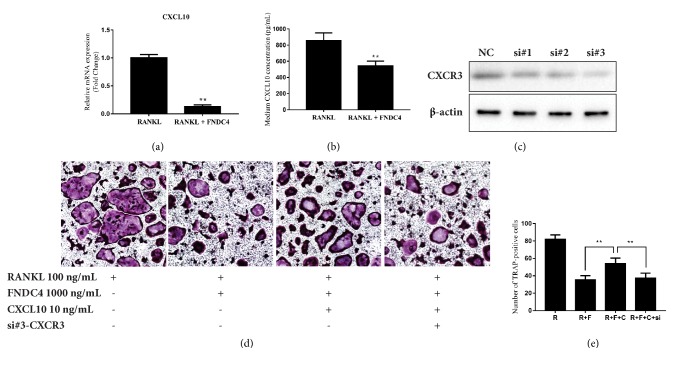
FNDC4 inhibited the osteoclast formation via downregulation of CXCL10. (a) FNDC4 could significantly suppress the expression of CXCL10 in BMMs. BMMs were treated with M-CSF (30 ng/mL) and RANKL (100 ng/mL) in the presence of absence of 1000 ng/mL FNDC4 for 2 days; then total RNA was collected for qPCR analysis. (b) BMMs were treated with M-CSF (30 ng/mL) and RANKL (100 ng/mL) in the presence of absence of 1000 ng/mL FNDC4 for 2 days; then the medium was collected to detect the concentration of CXCL10 using ELISA. (c) The selective silencing of CXCR3 was confirmed by western blotting; si#3 was selected for later experiments. (d, e) The addition of CXCL10 could attenuate the FNDC4-induced inhibition of osteoclast formation. BMMs were incubated with RANKL (100 ng/mL), M-CSF (30 ng/mL), and FNDC4 (1000 ng/mL) with or without the supplementation of CXCL10 (10 ng/mL) and siRNA against CXCR3 for 5 days. Representative images of TRAP-stained cells and quantitative analysis of TRAP-positive cells were presented for three independent experiments. Data were presented as *M* ± SD. ^*∗*^*p* < 0.05 and ^*∗∗*^*p* < 0.01.

As shown by RT-qPCR and ELISA analysis, CXCL10 mRNA expression was dramatically reduced by FNDC4 (Figure 6(a)); the release of this chemokine to medium also decreased significantly (Figure 6(b)). To determine whether FNDC4 inhibited osteoclast formation via downregulation of CXCL10, we added 10 ng/mL of CXCL10 in the presence of RANKL and FNDC4. The reduction of TRAP-positive number of osteoclasts by FNDC4 was partly ameliorated by the supplementation of CXCL10 (Figure 6(d)). In order to confirm the effect of CXCL10 in the FNDC4 inhibition of RANKL-mediated osteoclast maturation, we used siRNA against CXCR3 to knock down the expression of mRNA of CXCR3, and we used TRAP staining as a read-out assay. The selective silencing effect of siRNA was confirmed by western blotting (Figure 6(c)). According to the results in Figure 6(e), we validated that CXCL10/CXCR3 was involved in the FNDC4-induced suppression of osteoclast maturation.

## 4. Discussion

Osteoporosis is a widespread chronic condition linked to aging and with important health and socioeconomic consequences, whereas bone density can be increased and fracture risk reduced by drugs that inhibit bone resorption [18]. Considering the fact that osteoclasts are primarily responsible for bone resorption, potentially new drugs that targeted osteoclasts remain as one of the key candidates for the treatment of osteoporosis and other osteoclast-related osteolytic diseases. In our current study we found that FNDC4 could efficiently inhibit osteoclast formation and reduce bone resorption without a cytotoxic effect on BMMs. Furthermore, the findings demonstrated that FNDC4 could inhibit the RANKL-induced osteoclasts formation by suppressing NF-*κ*B signaling pathways and downregulating the expression of CXCL10.

FNDC4 is a member of the fibronectin type III domain family of proteins which shows high homology with irisin [10], and irisin is a newly identified exercise-related myokine that regulates energy expenditure by converting white fat tissue into brown fat [11]. An* in vitro* study by Colaianni et al. [19] has well characterized that irisin secreted from myoblast could directly target osteoblasts and thus enhance their differentiation; their subsequent* in vivo* study also showed intriguing results that injection of recombinant irisin in mice could induce significantly higher cortical bone mass along with higher bending strength [14]. Another experiment by Qiao and coworkers further proved that irisin could promote osteoblast proliferation and differentiation via the p38/MAPK signaling pathways [13]. Recently, Bosma and colleagues found that FNDC4 and FNDC5 had different expression profiles when comparing exercise and inflammation [10]. FNDC4 was not upregulated by exercise training, and FNDC5 remained downregulated or unchanged in three models of inflammation [10]. As osteoclast formation is characterized by the activation of inflammation-related pathways and no previous study has reported the effect of FNDC4 on osteoclast activation, we thus performed this* in vitro* study to evaluate the effect of FNDC4 in this process.

In our present study, we used BMMs as osteoclast precursor cells. The formation and function of osteoclasts were inhibited by FNDC4 in a dose-dependent manner. Consistent with these results, the gene expression of TRAP, CtsK, and NFATc1 that is increased during osteoclastogenesis which affects osteoclast function and fusion was also downregulated by FNDC4. Furthermore, we found that the treatment with FNDC4 could significantly suppress the transcriptional activity of NF-*κ*B signaling pathways, suggesting the potentially possible underlying mechanisms for the inhibitory effect of FNDC4 on osteoclast formation.

In order to identify gene signatures in FNDC4-treated BMMs and to use these to elucidate the underlying molecular mechanisms during osteoclast formation, we conducted a bioinformatics approach by downloading the GSE76172 gene expression profiling dataset from the Gene Expression Omnibus database. As shown by the Gene Ontology analysis, treatment of FNDC4 was associated with signal transduction, G-protein coupled receptor signaling pathway, immune system process, inflammatory response, cellular response to lipopolysaccharide, and so forth. By uploading the data of DEGs to STRING, we identified that CXCL10 was associated with the highest degree score, which was reported to positively regulate osteoclast differentiation [15, 20, 21]. As expected, CXCL10 mRNA expression in BMMs was also suppressed by the treatment of FNDC4 and the supplementation of CXCL10 could partially attenuate the inhibition of osteoclast formation by FNDC4.

## 5. Conclusion

Taken together, our study revealed that FNDC4 could inhibit the formation and function of osteoclast by suppressing the NF-*κ*B activity and downregulating CXCL10 expression. FNDC4 could be a novel regulator of osteoclast formation and has therapeutic potential for osteoporosis. The antiresorptive effect of FNDC4* in vivo* needs to be confirmed by future studies.
